# Empagliflozin and Cardiovascular Outcomes: A Systematic Review of Randomized Controlled Trials in Populations With Diabetes and Heart Failure

**DOI:** 10.7759/cureus.90150

**Published:** 2025-08-15

**Authors:** Farrukh Ansar, Abdullah Azzam, Usman Zafar, Sabeeh Ahmed, Fatima K Ghauri, AbdulRehman M Khan, Abdul Rafay Mehmood Khan, Muhammad Umair Khalil, Hamdah Iftikhar, Abdul Rehman Faiz, Muhammad Bilal Ahmad

**Affiliations:** 1 Department of Medicine, Alkhidmat Raazi Hospital, Rawalpindi, PAK; 2 Department of Neurology, National Hospital for Neurology and Neurosurgery, London, GBR; 3 Department of Medicine, Bahawal Victoria Hospital, Bahawalpur, PAK; 4 Department of Psychiatry, Oxleas NHS Foundation Trust, London, GBR; 5 Department of Medicine, Hazrat Bari Imam Sarkar (HBS) General Hospital, Islamabad, PAK; 6 Department of Emergency Medicine, Quaid-e-Azam International Hospital, Islamabad, PAK; 7 Department of Medicine, Quaid-e-Azam International Hospital, Islamabad, PAK

**Keywords:** a systematic review, cardiovascular outcomes, empagliflozin, heart failure, type 2 diabetes

## Abstract

Cardiovascular disease is a principal cause of morbidity and mortality worldwide, disproportionately affecting individuals with type 2 diabetes mellitus (T2DM) and heart failure. Empagliflozin, a sodium-glucose cotransporter 2 inhibitor, has shown promising cardioprotective effects beyond its glucose-lowering properties. This systematic review was conducted according to Preferred Reporting Items for Systematic Reviews and Meta-Analyses (PRISMA) guidelines, involving a comprehensive literature search of PubMed, Scopus, Embase, and Cochrane Central Register of Controlled Trials (CENTRAL) from inception to May 2025. Randomized controlled trials evaluating empagliflozin in adult patients with T2DM or heart failure and reporting cardiovascular outcomes were included. A total of 13 RCTs comprising 21,669 participants were synthesized, encompassing a broad spectrum of populations, including high-risk T2DM, chronic heart failure with reduced ejection fraction (HFrEF) and heart failure with preserved ejection fraction (HFpEF), and post-myocardial infarction cohorts. Empagliflozin was consistently associated with significant reductions in major adverse cardiovascular events, notably a 14-38% reduction in cardiovascular mortality and a 23-35% reduction in heart failure hospitalizations across key trials, such as EMPA-REG OUTCOME, EMPEROR-Reduced, and EMPEROR-Preserved. Smaller mechanistic studies highlighted additional benefits, including improvements in left ventricular function, cardiac remodeling, exercise capacity, and hemodynamic parameters. The safety profile of empagliflozin was favorable, with the main adverse event being an increased incidence of mild genital infections, and no excess in severe adverse or hypoglycemic events. Despite some heterogeneity in trial populations and follow-up, current evidence robustly supports empagliflozin as an effective and safe strategy to reduce cardiovascular risk in patients with T2DM and heart failure. Further research is warranted to explore long-term outcomes and expand indications to broader patient populations.

## Introduction and background

Cardiovascular disease (CVD) remains the foremost cause of morbidity and mortality globally, constituting a significant public health challenge [1]. In 2021, about 80% of the 20.5 million deaths related to cardiovascular disease occurred in low- and middle-income countries [1]. Globally, cardiovascular disease impacts 32.2% of individuals with type 2 diabetes mellitus (T2DM) [2]. Type 2 diabetes represents 90-95% of all diabetes cases worldwide [3]. In 2021, approximately 537 million individuals were living with diabetes, with projections indicating an increase to 643 million by 2030 and 783 million by 2045 [4]. Additionally, around 541 million people were estimated to have impaired glucose tolerance in 2021 [4]. It is further estimated that more than 6.7 million deaths among adults aged 20-79 years will be attributed to diabetes-related complications in 2021 [4]. Studies have shown that patients with T2DM face elevated cardiovascular risk that is two to four times higher than those without diabetes, with the risk increasing as glycemic control worsens [5]. Diabetes is linked to a 75% higher mortality rate in adults, with cardiovascular disease contributing significantly to this excess mortality [6]. Despite rigorous glycemic control and comprehensive management of conventional cardiovascular risk factors, the residual risk of adverse cardiovascular events remains substantial in this cohort [7]. Consequently, there exists an imperative need for therapeutic agents that not only ameliorate hyperglycemia but also confer direct cardiovascular protection.

Sodium-glucose cotransporter 2 (SGLT2) inhibitors have emerged as a novel class of antihyperglycemic agents that modulate renal glucose reabsorption, thereby promoting glycosuria and reducing plasma glucose levels [8]. Among these, empagliflozin has garnered considerable attention due to its demonstrated cardiovascular benefits that extend beyond glycemic management [9]. The EMPA-REG OUTCOME trial was a pivotal randomized controlled trial (RCT) that evaluated the effect of empagliflozin in a cohort of patients with T2DM and established cardiovascular disease [10]. This trial demonstrated a significant reduction in the composite primary outcome of cardiovascular death, nonfatal myocardial infarction, or nonfatal stroke, driven predominantly by a remarkable 38% relative risk reduction in cardiovascular mortality and a 35% reduction in hospitalization for heart failure [10]. These findings represented a paradigm shift in the treatment of patients with T2DM, emphasizing the potential of empagliflozin as a cardioprotective agent.

Subsequent investigations have expanded the therapeutic applicability of empagliflozin to patients with heart failure irrespective of diabetic status. The EMPEROR-Reduced trial, a landmark study in this context, enrolled patients with heart failure with reduced ejection fraction (HFrEF) and demonstrated that empagliflozin significantly reduced the composite endpoint of cardiovascular death or hospitalization for heart failure by 25%, with consistent effects observed in both diabetic and non-diabetic subpopulations [11]. This broadening of the therapeutic scope underscores the multifaceted mechanisms of empagliflozin that transcend glycemic control and impact cardiac physiology directly.

Moreover, analyses of early treatment effects have indicated that empagliflozin’s cardiovascular benefits manifest rapidly, with significant reductions in heart failure hospitalizations and cardiovascular mortality [12]. This early onset of effect, coupled with a generally favorable safety profile characterized by low hypoglycemia risk and manageable adverse events, substantiates the suitability of empagliflozin for integration into therapeutic regimens aimed at mitigating cardiovascular risk [13].

Given the accumulating body of evidence, a comprehensive synthesis of the cardiovascular outcomes associated with empagliflozin is warranted. This systematic review aimed to critically appraise and integrate findings from large-scale clinical trials to delineate the cardiovascular efficacy of empagliflozin. By synthesizing these results, we aim to inform clinical practice and guideline development, thereby improving cardiovascular risk management in patients with T2DM and heart failure.

## Review

Methods

Information Sources and Search Strategy

This systematic review was conducted in line with the Preferred Reporting Items for Systematic Reviews and Meta-Analyses (PRISMA) guidelines to ensure methodological rigor and transparency. Prior to commencing the review, a protocol was developed to outline the research objectives, inclusion and exclusion criteria, and the planned approach to data extraction and analysis. A comprehensive search of PubMed, Scopus, Embase, and the Cochrane Central Register of Controlled Trials (CENTRAL) was performed from database inception through May 2025. The PubMed search strategy utilized a combination of relevant keywords and Medical Subject Headings (MeSH) terms. ("Empagliflozin"[Mesh] OR "Sodium-Glucose Transporter 2 Inhibitors"[Mesh] OR empagliflozin[tiab] OR SGLT2[tiab]) AND ("Heart Failure"[Mesh] OR "Cardiovascular Diseases"[Mesh] OR "Diabetes Mellitus, Type 2"[Mesh] OR heart failure[tiab] OR cardiovascular[tiab] OR diabetes[tiab]) AND ("Randomized Controlled Trial"[Publication Type]) NOT ("Kidney Diseases"[Mesh] OR kidney[tiab] OR renal[tiab]). Reference lists of all eligible studies and recent reviews were also screened to identify additional relevant publications. Studies were included if they were randomized controlled trials evaluating empagliflozin and reporting cardiovascular outcomes in adults with heart failure or type 2 diabetes. In total, 13 RCTs meeting these criteria were identified and included in the qualitative synthesis.

Eligibility Criteria

We only included studies that were randomized controlled trials and specifically looked at the cardiovascular effects of empagliflozin in adults. To be eligible, the participants had to be 18 years or older and diagnosed with either type 2 diabetes, heart failure with HFrEF, or coronary artery disease (CAD). We considered studies that tested empagliflozin at any dose or for any length of time, as long as it was compared to either a placebo or standard care without empagliflozin. To make sure the studies were relevant, they needed to report at least one cardiovascular outcome, such as cardiovascular death, hospitalizations for heart failure, changes in heart structure or function, levels of heart biomarkers like N-terminal pro-B-type natriuretic peptide (NT-proBNP), or assessments of physical ability like the 6-minute walk test. We only reviewed articles published in English. We did not include review papers, editorials, conference summaries without full data, animal studies, or any research that didn’t report on cardiovascular outcomes.

Study Selection

All the titles and abstracts retrieved in the search were independently reviewed by two separate teams, each consisting of three members. Both teams used the predefined eligibility criteria to decide which studies should move forward. If a study clearly met the criteria or there was any uncertainty about its eligibility, it was selected for a full-text review. Next, the same teams independently assessed the full-text articles to confirm if they met the inclusion criteria. Whenever there were any ambiguities or disagreements, the teams discussed the issues together. If they were unable to reach a consensus, the principal author stepped in to help resolve the matter.

Data Collection Process

Data extraction were carried out independently by two separate teams, each consisting of two members. Both teams used a standardized form created specifically for this review, which collected important details, such as study identifiers, trial design, participant characteristics, intervention and comparator details, length of follow-up, and the cardiovascular outcomes reported. After initial extraction, both teams cross-checked each other’s data sheets to make sure everything was accurate and complete. If there were any discrepancies or ambiguities, these were discussed among the team members, and the principal author made the final decision whenever needed.

Data Items

The data we collected covered a wide range of study details. This included basic information like the study design, the number of participants, and key demographic details, such as age, sex, diabetes status, heart failure status, and baseline heart function. We also recorded information about the treatment. Specifically, the dose of empagliflozin used, how long participants received it, and what it was compared against. For outcomes, we looked at several important cardiovascular results, including deaths due to cardiovascular causes, deaths from any cause, hospitalizations for heart failure, composite cardiovascular events, changes in heart structure and function, heart biomarkers, and measures of physical ability.

Data Synthesis and Analysis

Given the clinical heterogeneity across studies with respect to patient populations, outcome measures, and follow-up durations, a quantitative meta-analysis was not feasible. Therefore, a qualitative narrative synthesis was undertaken. Key findings were organized and summarized in tables according to outcome domains, such as mortality, heart failure hospitalization, cardiac remodeling, functional capacity, and safety. Effect estimates, including hazard ratios, mean differences, confidence intervals, and p-values, were extracted and reported to provide a comprehensive summary of the evidence.

Results

**Figure 1:**
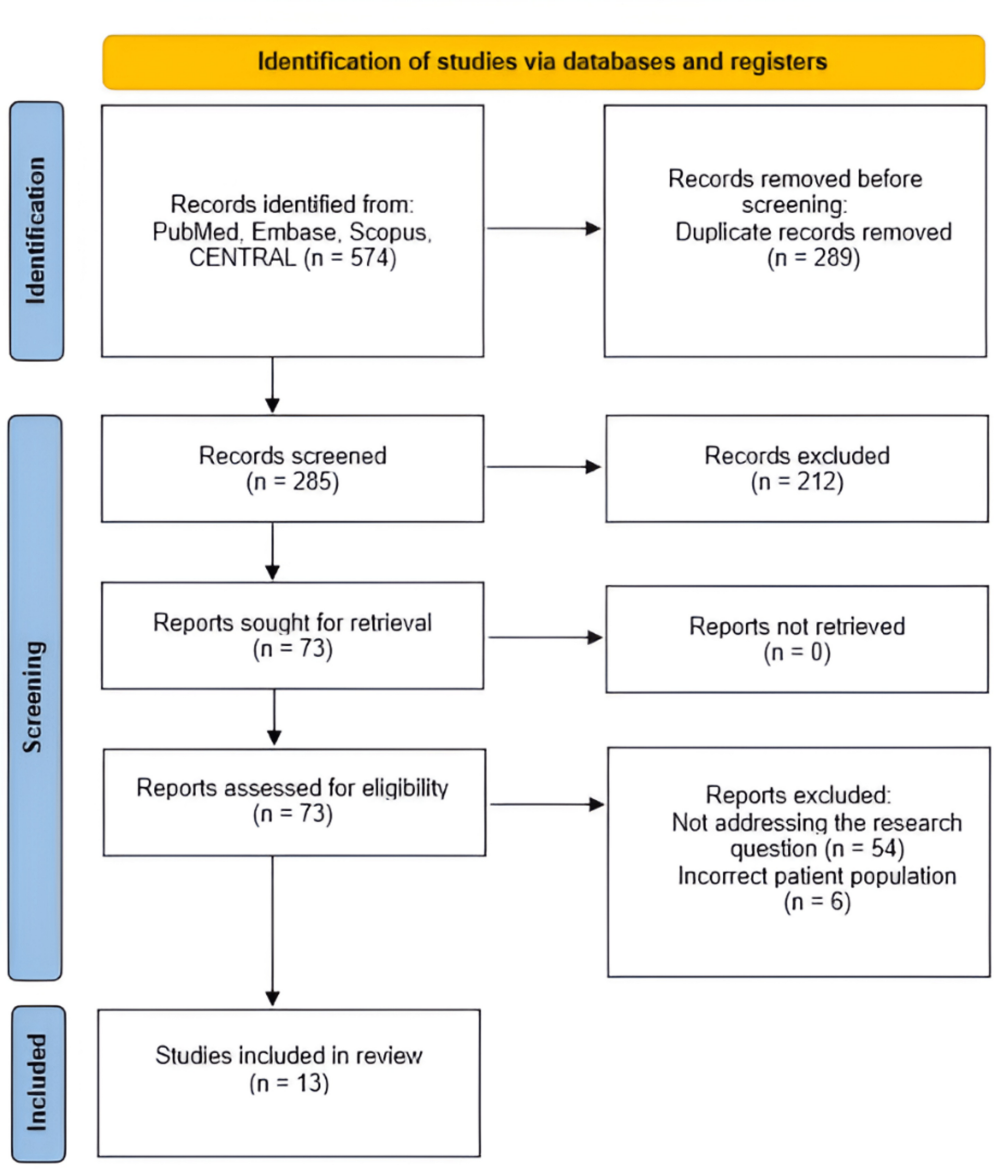
PRISMA flow chart illustrating the study identification and selection process. PRISMA: Preferred Reporting Items for Systematic Reviews and Meta-Analyses; CENTRAL: Cochrane Central Register of Controlled Trials

A total of 574 records were identified through systematic searches of PubMed, Embase, Google Scholar, and Scopus. After removing 289 duplicate records, 285 unique articles remained for screening. Titles and abstracts were reviewed, resulting in the exclusion of 212 articles that did not meet the inclusion criteria. The full texts of the remaining 73 articles were then retrieved and assessed for eligibility. Following the application of predefined inclusion and exclusion criteria, 60 articles were excluded; 54 for not addressing the research question and six for including an incorrect patient population. Ultimately, 13 randomized controlled trials were included in the final analysis Figure 1.

This systematic review included 13 randomized controlled trials evaluating the cardiovascular effects of empagliflozin across a broad spectrum of patient populations (n=21,669). The largest trial, EMPA-REG OUTCOME, enrolled 7,020 patients with T2DM at high cardiovascular risk and assessed empagliflozin’s impact on major adverse cardiovascular events over 3.1 years [10]. The EMPEROR-Reduced and EMPEROR-Preserved trials evaluated empagliflozin in patients with chronic HFrEF and HFpEF [11], respectively, including over 3,700 and 5,900 participants with follow-up periods up to 26 months [14].

Empagliflozin’s efficacy was also assessed in specific clinical scenarios as follows: EMPACT-MI investigated its use in over 3,200 patients following acute myocardial infarction [15], while EMPULSE focused on those hospitalized with acute heart failure [16]. Several mechanistic and smaller-scale studies provided additional insights into empagliflozin’s effects on cardiac structure and function. EMPA-TROPISM included patients with non-diabetic HFrEF [17], SUGAR-DM-HF enrolled individuals with T2DM or prediabetes and HFrEF [18], and EMPA-HEART CardioLink-6 studied T2DM patients with coronary artery disease [19]. RECEDE-CHF evaluated hemodynamic effects in chronic HFrEF [20], and the EMMY trial assessed outcomes following acute myocardial infarction [21].

Further, the EMBODY trial explored autonomic regulation in acute myocardial infarction (AMI) patients with T2DM [22], and the Empire HF trial enrolled patients with mild-symptom chronic HFrEF [23]. Lastly, a randomized controlled trial by Ovchinnikov et al. examined empagliflozin in 70 patients with HFpEF and T2DM, focusing on functional and hemodynamic outcomes [24]. Across these studies, empagliflozin was administered at doses of 10 or 25 mg daily, with placebo controls, and follow-up durations ranged from six weeks to over three years. The characteristics of these included studies, including trial names, populations, interventions, sample sizes, and follow-up durations, are summarized in Table 1.

**Table 1:** Characteristics of included randomized controlled trials evaluating cardiovascular outcomes of empagliflozin. HFrEF: heart failure with reduced ejection fraction; HFpEF: heart failure with preserved ejection fraction; T2DM: type 2 diabetes mellitus; AMI: acute myocardial infarction; EF: ejection fraction

Trial names	Studies	Population	Intervention vs. placebo	Sample size (n)	Follow-up duration
EMPA-REG OUTCOME [10]	Zinman et al. (2015)	T2DM patients, high cardiovascular risk	Empagliflozin 10/25 mg daily	7,020	3.1 years
EMPEROR-Reduced [11]	Packer et al. (2020)	Chronic HFrEF (EF ≤40%)	Empagliflozin 10 mg daily	3,730	16 months
EMPEROR-Preserved [14]	Anker et al. (2021)	Chronic HFpEF (EF ≥40%)	Empagliflozin 10 mg daily	5,988	26.2 months
EMPACT-MI [15]	Butler et al. (2024)	Post-AMI patients at risk for heart failure	Empagliflozin 10 mg daily	3,260	17.9 months
EMPULSE [16]	Voors et al. (2022)	Hospitalized acute HF (de novo or chronic)	Empagliflozin 10 mg daily	530	90 days
EMPA-TROPISM [17]	Santos-Gallego et al. (2021)	Non-diabetic HFrEF	Empagliflozin 10 mg daily	84	6 months
SUGAR-DM-HF [18]	Lee et al. (2021)	T2DM/prediabetes with HFrEF	Empagliflozin 10 mg daily	105	36 weeks
EMPA-HEART CardioLink-6 [19]	Verma et al. (2019)	T2DM and coronary artery disease	Empagliflozin 10 mg daily	97	6 months
RECEDE-CHF [20]	Mordi et al. (2020)	T2DM and chronic HFrEF	Empagliflozin 25 mg daily	23	6 weeks
EMMY [21]	von Lewinski et al. (2022)	Post-acute myocardial infarction	Empagliflozin 10 mg daily	476	26 weeks
EMBODY [22]	Shimizu et al. (2020)	AMI patients with T2DM	Empagliflozin 10 mg daily	96	24 weeks
Empire HF [23]	Jensen et al. (2020)	Chronic mild-symptom HFrEF	Empagliflozin 10 mg daily	190	12 weeks
Ovchinnikov RCT [24]	Ovchinnikov et al. (2025)	HFpEF with T2DM	Empagliflozin 10 mg daily	70	6 months

Cardiovascular mortality and hospitalizations for heart failure

In the EMPEROR-Reduced trial (n=3,730; median follow-up 16 months), empagliflozin significantly decreased the composite endpoint of cardiovascular death or hospitalization for heart failure compared to placebo (hazard ratio {HR}: 0.75; 95% confidence interval {CI}: 0.65-0.86; p<0.001) [11]. This corresponds to a 25% relative risk reduction, indicating that empagliflozin substantially lowered the likelihood of experiencing these serious outcomes over the follow-up period. The primary driver of this benefit was a marked reduction in total hospitalizations for heart failure (HR: 0.70; 95% CI: 0.58-0.85; p<0.001), reflecting a 30% lower risk relative to placebo. This finding suggests that empagliflozin not only reduces first events but also has a robust impact on recurrent heart failure hospitalizations, which are associated with significant morbidity. Similarly, in patients with heart failure with HFpEF, the EMPEROR-Preserved trial (n=5,988; median follow-up 26 months) demonstrated a significant 21% relative reduction in the combined risk of cardiovascular death or hospitalization for heart failure compared to placebo (HR: 0.79; 95% CI: 0.69-0.90; p<0.001) [14]. This means that, over the course of the study, patients receiving empagliflozin were notably less likely to experience these serious outcomes.

In patients with T2DM at high cardiovascular risk, the EMPA-REG OUTCOME trial (n=7,020; median follow-up 3.1 years) provided robust evidence for the cardioprotective effects of empagliflozin [10]. Treatment with empagliflozin resulted in a 14% relative reduction in the risk of the primary composite outcome of cardiovascular death, nonfatal myocardial infarction, or nonfatal stroke compared to placebo (HR: 0.86; 95% CI: 0.74-0.99; p=0.04), meaning that fewer patients experienced major adverse cardiovascular events during follow-up. Notably, empagliflozin produced a 38% reduction in the risk of cardiovascular mortality alone (HR: 0.62; 95% CI: 0.49-0.77; p<0.001), indicating a substantial and clinically meaningful impact on survival. The trial also showed a 35% reduction in the risk of hospitalization for heart failure (HR: 0.65; 95% CI: 0.50-0.85; p=0.002), translating to significantly fewer patients requiring admission for heart failure during the study period. To further summarize the cardiovascular benefits observed, Table 2 details primary endpoints, hazard ratios, confidence intervals, and statistical significance from each trial, enabling direct comparison of outcomes across studies.

**Table 2:** Summary of major cardiovascular outcomes with empagliflozin in included trials. CV: cardiovascular; HHF: hospitalization for heart failure; MI: myocardial infarction; LVEDV: left ventricular end-diastolic volume; LVESV: left ventricular end-systolic volume; NT-proBNP: N-terminal pro-B-type natriuretic peptide; QoL: quality of life; NS: not significant.

Trial name	Primary cardiovascular endpoint	Hazard ratio (95% CI)/key findings	p-Value
EMPA-REG OUTCOME [10]	CV death, nonfatal MI, or nonfatal stroke	HR: 0.86 (0.74-0.99); CV death: HR: 0.62 (0.49-0.77)	0.04; <0.001
EMPEROR-Reduced [11]	CV death or HHF	HR: 0.75 (0.65-0.86)	<0.001
EMPEROR-Preserved [14]	CV death or HHF	HR: 0.79 (0.69-0.90)	<0.001
EMPACT-MI [15]	All-cause death or HHF	HR: 0.90 (0.76-1.06); HHF alone HR: 0.77 (0.60-0.98)	0.21; 0.031
EMPULSE [16]	Clinical benefit (mortality, HF events, QoL)	Win ratio: 1.36 (1.09-1.68)	0.0054
EMPA-TROPISM [17]	Reduction in LVEDV/LVESV	LVEDV (-25.1 mL vs. -1.5 mL); LVESV (-26.6 mL vs. -0.5 mL)	<0.001
SUGAR-DM-HF [18]	Reduction in LVESV	-6.0 mL/m² reduction vs. placebo	0.015
EMPA-HEART CardioLink-6 [19]	Reduction in left ventricular mass index	-3.35 g/m² reduction vs. placebo	0.01
RECEDE-CHF [20]	Increase in 24-hour urine volume	+545 mL/day increase vs. placebo	0.005
EMMY [21]	NT-proBNP reduction	-15% reduction vs. placebo	0.026
EMBODY [22]	Heart rate variability markers	Improved in empagliflozin; no significant intergroup difference	NS
Empire HF [23]	NT-proBNP reduction	No significant reduction vs. placebo	NS
Ovchinnikov RCT [24]	Improvement in exercise capacity (6-min walk distance)	Significant improvement vs. placebo	<0.05

Acute myocardial infarction and subsequent heart failure risk

Several trials have evaluated the effects of empagliflozin when initiated soon after acute myocardial infarction. In the EMPACT-MI trial (n=6,522; median follow-up 17.9 months), empagliflozin did not significantly reduce the risk of the composite endpoint of HHF or all-cause death compared to placebo (HR: 0.90; 95% CI: 0.76-1.06; p=0.21), indicating no clear difference in these major outcomes within the studied timeframe [15]. However, a 23% reduction in the risk of heart failure hospitalization alone was observed (HR: 0.77; 95% CI: 0.60-0.98; p=0.031), suggesting that empagliflozin may help prevent heart failure-related admissions following myocardial infarction, even if it does not significantly impact overall mortality in this context.

Complementing these findings, the EMMY trial (n=476; follow-up 26 weeks) demonstrated significant improvements in markers of cardiovascular health [21]. Specifically, empagliflozin use resulted in a 15% reduction in NT-proBNP levels (p=0.026), a biomarker of cardiac stress, and a modest increase in LVEF by 1.5% (p=0.029).

Cardiac remodeling and left ventricular function

Several trials have specifically evaluated the effects of empagliflozin on cardiac structure and LV function. In the EMPA-TROPISM trial (n=84; six-month follow-up), empagliflozin significantly reduced LV end-diastolic and end-systolic volumes (LVEDV: -25.1 mL vs. -1.5 mL; LVESV: -26.6 mL vs. -0.5 mL; both p<0.001), indicating that patients experienced substantial decreases in the size of the heart chambers at the end of filling and contraction [17]. Additionally, empagliflozin increased LVEF by 6.0% (p<0.001) compared to placebo, reflecting a marked improvement in the heart’s pumping ability.

Similarly, in the SUGAR-DM-HF trial (n=105; 36-week follow-up), empagliflozin significantly reduced LV end-systolic volume indexed to body surface area by 6.0 mL/m² (p=0.015) [18]. This reduction means the heart was able to contract more effectively, leaving less blood in the ventricle after each beat.

The EMPA-HEART CardioLink-6 trial (n=97; six-month follow-up) also demonstrated a reduction in LV mass index by 3.35 g/m² (p=0.01), suggesting a reversal of harmful cardiac remodeling [19]. Collectively, these findings indicate that empagliflozin not only improves cardiac structure but also enhances LV function, which could translate into better long-term outcomes for patients with heart failure.

Functional capacity and quality of life

In the EMPULSE trial (n=530; 90-day follow-up), empagliflozin was associated with a substantial clinical benefit, as evidenced by a win ratio of 1.36 (95% CI: 1.09-1.68; p=0.0054) for the composite endpoint of mortality, heart failure events, and quality-of-life measures [16]. This indicates that, for every 100 patient pairs compared, those receiving empagliflozin were 36% more likely to have a better overall clinical outcome than those on placebo.

Similarly, the EMPA-TROPISM trial demonstrated marked improvements in physical performance, with patients in the empagliflozin group walking an average of 81 m farther in the 6-minute walk test, compared to a decrease of 35 m in the placebo group (p<0.001) [22]. This translates to a notable enhancement in functional status and exercise capacity.

In a randomized controlled open-label trial by Ovchinnikov et al., 70 patients with type 2 diabetes mellitus and heart failure with HFpEF were assigned to receive either empagliflozin (10 mg/day) or standard care for six months [24]. Patients treated with empagliflozin experienced a significant improvement in functional capacity, as demonstrated by a greater increase in the distance walked during the 6-minute walk test (6MWD) compared to the control group (p<0.05). Additionally, reductions in left atrial (LA) volume index and the E/e′ ratio, both at rest and during exercise, were observed in the empagliflozin group (p<0.05).

Additional hemodynamic and biochemical outcomes

Empagliflozin demonstrated favorable hemodynamic effects that extend beyond heart failure outcomes. In the RECEDE-CHF crossover trial (n=23; six-week phases), empagliflozin treatment led to a significant increase in urinary volume by 545 mL (p=0.005), without a corresponding rise in sodium excretion [20]. This suggests that empagliflozin promotes diuresis through mechanisms other than simply increasing sodium loss, which may have unique implications for volume management in heart failure patients.

The EMBODY trial (n=96; 24-week follow-up) evaluated the drug’s impact on autonomic regulation following acute myocardial infarction [22]. Empagliflozin significantly improved cardiac autonomic function, as evidenced by an increase in the standard deviation of all 5-minute mean RR intervals (SDANN: +11.6 ms, p=0.02), indicating greater variability in heart rate. It also reduced the low-frequency to high-frequency (LF/HF) ratio (-0.57, p=0.01), reflecting a shift towards parasympathetic dominance. Notably, improvement in heart rate turbulence (HRT, p=0.01), an indicator of autonomic responsiveness and cardiovascular risk, was observed only in the empagliflozin group.

Safety and tolerability

Empagliflozin was generally well-tolerated across the reviewed trials, with the primary safety concern consistently being an increased incidence of uncomplicated genital infections. Specifically, the EMPEROR-Reduced trial reported genital tract infections more frequently in the empagliflozin group compared with placebo (1.7% vs. 0.6%) [11], although these events were mostly mild or moderate and manageable without treatment discontinuation [10]. Similarly, in the EMPA-REG OUTCOME trial, genital infections were significantly higher in the empagliflozin group than placebo (6.4% vs. 1.8%; p<0.001), with no associated increase in other adverse cardiovascular or renal events [10].

Other trials supported a similarly reassuring safety profile. The EMPACT-MI trial, involving patients with post-acute myocardial infarction, noted comparable adverse event rates between the empagliflozin and placebo groups, without significant differences in severe or serious adverse events [15]. The EMPULSE trial reported fewer serious adverse events in empagliflozin-treated patients compared to placebo (32.3% vs. 43.6%), suggesting good tolerability even when initiated in acute heart failure settings [16].

Cardiovascular-specific safety was affirmed by the EMPEROR-Preserved trial, which noted no increased risks of hypotension, hypovolemia, or electrolyte abnormalities associated with empagliflozin [14]. Similarly, smaller trials, such as EMPA-TROPISM [17], EMMY [21], and EMBODY, consistently found no increase in severe cardiovascular adverse events with empagliflozin relative to placebo [22].

Discussion

Empagliflozin, a SGLT2 inhibitor, has demonstrated significant cardiovascular benefits in patients with T2DM. The EMPA-REG OUTCOME trial revealed a 38% relative risk reduction in CV mortality and a 35% reduction in HHF among patients receiving empagliflozin compared to placebo [10]. These findings were shown by subsequent studies, including the EMPEROR-Reduced trial, which reported a 25% reduction in the combined risk of cardiovascular death or hospitalization for heart failure in patients with HFrEF, irrespective of diabetes status [11]. A meta-analysis of RCTs further supports these results; it assessed 23,344 participants, with 12,849 receiving empagliflozin. Treatment with empagliflozin led to a significant reduction in cardiovascular mortality (HR: 0.86; 95% CI: 0.78-0.96). Additionally, empagliflozin was associated with a 30% lower risk of hospitalization for heart failure (HR: 0.70; 95% CI: 0.64-0.77) in all included studies. Sensitivity analyses supported the reliability of these findings [25].

The cardiovascular benefits of empagliflozin are attributed to several mechanisms beyond its glucose-lowering effects. While the precise mechanisms underlying these benefits are multifactorial and not yet fully elucidated, emerging evidence highlights several key pathways directly impacting cardiovascular structure and function.

First, empagliflozin induces a sustained osmotic diuresis and natriuresis, resulting in modest but clinically meaningful reductions in intravascular volume and preload [25]. This volume unloading effect is particularly advantageous in patients with heart failure, where congestion and elevated cardiac filling pressures are central contributors to morbidity [26]. By lowering preload and, through its modest antihypertensive action, reducing afterload, empagliflozin effectively attenuates myocardial wall stress and oxygen demand [25,26]. This hemodynamic optimization is complemented by a consistent reduction in systolic and diastolic blood pressure, an effect achieved without compensatory increases in heart rate, which further alleviates cardiac workload and supports ventricular function [27].

Empagliflozin’s impact on vascular biology is also of great significance. The agent has been demonstrated to reduce arterial stiffness and enhance endothelial function [28]. These are the two interrelated determinants of vascular compliance and myocardial perfusion. Improved endothelial-dependent vasodilation is thought to be mediated by decreased oxidative stress and inflammation, as well as enhanced nitric oxide bioavailability [29]. These vascular effects translate into improved coronary flow reserve, supporting myocardial oxygen delivery, particularly in patients with established atherosclerotic disease or microvascular dysfunction [29].

Additionally, empagliflozin favorably influences a host of metabolic parameters that have downstream cardiovascular effects. Notably, modest but consistent reductions in body weight and visceral adiposity are observed with SGLT2 inhibitor therapy [30]. These changes in body composition, coupled with improved glycemic control and lower levels of circulating insulin, may mitigate the progression of adverse cardiac remodeling and contribute to the observed reductions in heart failure hospitalization and cardiovascular mortality seen in clinical trials [31]. Empagliflozin has also been associated with a reduction in serum uric acid and improvements in markers of systemic inflammation, both of which have established roles in cardiovascular pathophysiology. Empagliflozin reduces serum uric acid levels primarily by promoting uricosuria; inhibition of SGLT2 in the renal proximal tubule increases glucose excretion, which, through competitive interaction with urate transporters, enhances the elimination of uric acid in the urine [32]. Concurrently, empagliflozin improves markers of systemic inflammation, a benefit attributed to its capacity to decrease visceral adiposity, enhance insulin sensitivity, and directly suppress pro-inflammatory cytokines, such as C-reactive protein and interleukin-6 [33]. As both hyperuricemia and chronic inflammation are well-established contributors to endothelial dysfunction, atherosclerosis, and adverse cardiac remodeling, these pleiotropic effects of empagliflozin provide mechanistic insight into its observed cardiovascular benefits beyond glycemic control [34].

At the myocardial level, preclinical studies suggest that empagliflozin may shift cardiac substrate utilization from glucose and free fatty acids toward ketone bodies, which are a more energy-efficient fuel source [35]. This metabolic reprogramming may improve myocardial energetics, reduce oxidative stress, and enhance contractile efficiency, particularly in the context of heart failure, where energy starvation is a hallmark [36]. Collectively, the cardiovascular benefits of empagliflozin are likely the result of this integrated impact on preload, afterload, vascular function, metabolic effects, and potentially myocardial energetics, offering a compelling therapeutic option that addresses multiple pathophysiological aspects in cardiovascular disease.

Empagliflozin is generally considered safe and well-tolerated, with a safety profile established across meta-analyses involving diverse populations [37]. Empagliflozin reduces glucose reabsorption in the renal proximal tubule, leading to increased urinary glucose excretion [38]. This creates a glucose-rich environment in the lower urinary tract, which predisposes individuals to genital mycotic infections and, to a lesser degree, urinary tract infections [38]. The diuretic effect can also lead to volume depletion, especially in elderly or frail patients, but serious events are rare with appropriate clinical monitoring [38].

The incidence of genital infections is increased two- to four-fold with empagliflozin compared to placebo. In the EMPA-REG OUTCOME trial (n=7,020), genital infections occurred in approximately 6.4% of patients receiving empagliflozin vs. 1.8% on placebo (p<0.001) [10]. The EMPA-REG OUTCOME trial reported UTI rates of 1.8% (empagliflozin group) vs. 1.7% in the placebo group [10]. Another pooled analysis from 20 trials reported incidence of UTI-related events were comparable between the empagliflozin group and the placebo group, at 9.27 and 9.70 events per 100 patient-years, respectively [38]. In a meta-analysis by Zhang et al. involving 15 RCTs and 7,891 patients, empagliflozin demonstrated a favorable safety and tolerability profile [39]. The incidence of hypoglycemia and UTIs was found to be comparable between the empagliflozin and placebo groups, indicating that empagliflozin does not significantly increase the risk of these events. Additionally, a reduced risk of hyperglycemia was observed in the empagliflozin group (risk ratio: 0.34; 95% CI: 0.23-0.49; p<0.00001), supporting its glycemic control benefits. However, the study reported a significantly higher risk of genital infections in patients treated with empagliflozin compared to placebo (risk ratio: 2.59; 95% CI: 1.80-3.71; p<0.00001) [39].

In summary, empagliflozin demonstrates a generally well-tolerated safety profile, with most adverse events being mild and manageable. While the risk of genital infections is notably higher, particularly among patients predisposed to mycotic infections, the incidence of urinary tract infections and hypoglycemia remains comparable to placebo. Volume depletion may occur due to the drug’s osmotic diuretic effect, especially in elderly or frail individuals, but serious complications are uncommon with appropriate clinical oversight. When weighed against its glycemic, cardiovascular, and renal benefits, the overall risk-benefit profile of empagliflozin remains highly favorable for patients with type 2 diabetes mellitus [40].

Limitations and future directions

Despite the comprehensive evaluation of 13 RCTs encompassing diverse patient populations, several limitations must be acknowledged. First, heterogeneity exists among the included studies with respect to patient selection criteria, baseline comorbidities, and the spectrum of heart failure phenotypes (HFrEF vs. HFpEF), which may impact the generalizability of findings to all patient subgroups. The follow-up durations varied substantially, from six weeks to over three years, limiting conclusions about the long-term durability of empagliflozin’s cardiovascular benefits and safety outcomes. Furthermore, the majority of studies enrolled patients with established cardiovascular disease or high-risk profiles, potentially reducing the external validity of results to lower-risk or primary prevention populations. Another important limitation is the underrepresentation of certain demographic groups, such as older adults, women, and ethnic minorities, which constrains our ability to evaluate the consistency of empagliflozin’s effects across these subpopulations. Some trials also had relatively small sample sizes or were conducted in single centers, limiting the statistical power to detect rare adverse events or subgroup effects. While most studies reported favorable safety outcomes, adverse event reporting was not always standardized or sufficiently granular, particularly for less common but clinically relevant complications. Moreover, mechanistic insights remain largely speculative, as the majority of trials focused on clinical endpoints without dedicated mechanistic substudies or biomarker-driven analyses.

Future research should prioritize large-scale, pragmatic trials and real-world cohort studies to assess the effectiveness, safety, and cost-effectiveness of empagliflozin in broader, more heterogeneous populations, including those with earlier stages of heart failure or at lower cardiovascular risk. Longer-term studies are needed to establish the persistence of cardiovascular and safety benefits, and to evaluate outcomes beyond three years of therapy. Future investigations should also focus on elucidating the molecular and physiological mechanisms underlying empagliflozin’s cardioprotective effects, employing advanced imaging, omics technologies, and comprehensive biomarker profiling. Special attention should be given to underrepresented groups and the potential influence of concomitant therapies, coexisting renal impairment, and polypharmacy. Finally, head-to-head comparisons of empagliflozin with other SGLT2 inhibitors, as well as integration into multi-drug regimens for heart failure and diabetes, will help clarify its optimal role in contemporary clinical practice. Collectively, these future directions will contribute to more individualized, evidence-based application of empagliflozin in cardiovascular medicine.

## Conclusions

This review provides evidence that empagliflozin delivers significant and consistent reductions in heart failure hospitalizations and cardiovascular mortality among a diverse range of high-risk populations, including individuals with type 2 diabetes, heart failure with both reduced and preserved ejection fraction, and those with recent myocardial infarction. The cardiovascular advantages of empagliflozin are likely attributable to a convergence of mechanisms that extend beyond glycemic control, encompassing hemodynamic optimization, reverse cardiac remodeling, and beneficial effects on vascular function and metabolic parameters. Notably, the safety profile of empagliflozin remains highly favorable, with the majority of adverse events being mild and easily managed, thereby reinforcing its suitability for widespread clinical use. Nevertheless, ongoing research is essential to clarify the durability of these benefits over longer treatment durations, to deepen our understanding of the underlying biological pathways, and to define empagliflozin’s optimal role within the evolving landscape of personalized cardiovascular and heart failure therapy.
